# Supplementary material for: Physiological specialization of the brain in bumble bee castes: Roles of dopamine in mating-related behaviors in female bumble bees
Source: PLoS One. 2024 Mar 13;19(3):e0298682. doi: 10.1371/journal.pone.0298682 (PMC10936820; doi:10.1371/journal.pone.0298682)
Supplement: S1 Table — (PDF) [file pone.0298682.s001.pdf]

S1 Table. Primer sequence of the genes for RT-qPCR.

Protein type	Description	Gene	Forward primer	Reverse primer	Ref.
Receptor	Dopamine D1-like receptor 1 (Dop1)	*BigDop1*	TAACGAAACTGCCGGATACC	CTTGCAATATGAAGCGACGA	Sasaki et al. 2021
	Dopamine D1-like receptor 2 (Dop2)	*BigDop2*	GAGGGAGAAGCACGAAACTG	TGGTCGATCATTCACCAGAA	Sasaki et al. 2021
	Dopamine D2-like receptor (Dop3)	*BigDop3*	CAACTACGCCCGTCGATATT	ACGCTCTATATCCGCTCGAA	Sasaki et al. 2021
	Dopamine / Ecdysteroid receptor (DopEcR)	*BigDopEcR*	ATCTCGCGAAACGAAAAAGA	ACTCGCGAAGTCAAAGCAAT	This study
Reference	Actin-5C	*BigACT*	GATGGATGGTCCAGACTCGT	GAATCGCTGACAGAATGCAA	Sasaki et al. 2021
	Glyceraldehyde-3-phosphate dehydrogenase 2 (GAPDH)	*BigGAPDH*	CATTCCAGCCCTTAATGGAA	CTTCAAGGGTCCTTCAGCAG	Sasaki et al. 2021
	40S ribosomal protein S3	*BigRPS3*	CAACCCGTCATGTCCTTCTT	CGCAAGTGGTATTGGTTGTG	Sasaki et al. 2021
	40S ribosomal protein S49	*BigRPS49*	GAAGTTCATTCGTCATCAGAG	TTGTCCCTTAAATCGCCTAC	Kodaira et al. 2009
