# Supplementary material for: Physiological specialization of the brain in bumble bee castes: Roles of dopamine in mating-related behaviors in female bumble bees
Source: PLoS One. 2024 Mar 13;19(3):e0298682. doi: 10.1371/journal.pone.0298682 (PMC10936820; doi:10.1371/journal.pone.0298682)
Supplement: S2 Table — (PDF) [file pone.0298682.s002.pdf]

S2 Table. Data of behavioral activities in workers and gynes in bumble bees (Figure 2)

Locomotor activity

Day age	0		2		4		6		8	
Caste	Worker	Gyne	Worker	Gyne	Worker	Gyne	Worker	Gyne	Worker	Gyne
	20	0	10	10	59	99	0	56	0	54
	0	0	0	13	0	17	0	56	0	62
	0	0	0	1	0	49	0	79	1	56
	0	8	64	48	0	69	94	41	54	26
	0	2	73	0	87	57	0	46	0	47
	12	0	0	31	230	39	0	56	0	9
	40	0	0	15	14	58	0	60	5	41
	129	15	16	0	0	0	40	73	57	0
	0	1	75	0	0	81	0	48	0	54
	0	17	24	0	0	38	27	60	0	57
	19	0	9	0	0	80	0	26	19	73
	9	5		24	0	42	1	0		63
	17	0		0	0		69	0		13
	2	0		57			0	25		34
		7		73				0		73
		0		0				41		
		37		17				5		
		0						37		
		42						70		
		1						15		
		0						0		
								87		
mean	17.71429	6.428571	24.63636	17	30	52.41667	16.5	40.04545	12.36364	44.13333
SE	9.116721	2.638155	9.237545	5.520683	18.33555	8.064567	8.158963	5.897566	6.654148	5.953684
N	14	21	11	17	13	12	14	22	11	15

Light avoidance

Day age	0		2		4		6		8	
Caste	Worker	Gyne	Worker	Gyne	Worker	Gyne	Worker	Gyne	Worker	Gyne
	900	900	900	852	429	293	900	304	900	120
	900	900	900	862	699	113	900	375	900	704
	900	900	805	900	576	815	900	263	810	649
	875	899	899	144	881	90	900	743	900	365
	704	894	804	900	900	365	833	633	820	555
	900	900	900	365	900	614	900	326	900	880
	900	900	900	207	900	790	422	721	900	258
	900	855	884	900	900	900	900	566	900	900
	112	900	684	900	900	140	900	447	900	494
	831	728	47	900	900	863	756	417	900	142
	827	900	11	900	900	247	900	786	817	486
	900	900		900	900	744	900	900		735
	670	900		900	900		784	900		36
	900	900		492			900	689		703
		630		302				900		287
		900		900				753		
		756		478				855		
		900						798		
		292						705		
		900						397		
		900						900		
								577		
mean	801.3571	840.6667	703.0909	694.2353	821.9231	497.8333	842.5	634.3182	877	487.6
SE	56.77937	31.66103	102.5395	69.77235	42.99157	92.11824	34.83694	46.01258	11.89729	71.56814
N	14	21	11	17	13	12	14	22	11	15

Proportion of flying individuals

Caste	Worker				Gyne			
Day age	0	2	4	6	0	2	4	6
Level 1	72.72727	75	60	76.47059	83.33333	54.54545	40	30
Level 2	27.27273	0	0	0	16.66667	0	0	0
Level 3	0	25	40	23.52941	0	45.45455	60	70
N	8	12	10	17	10	11	10	10
