# Supplementary material for: Physiological specialization of the brain in bumble bee castes: Roles of dopamine in mating-related behaviors in female bumble bees
Source: PLoS One. 2024 Mar 13;19(3):e0298682. doi: 10.1371/journal.pone.0298682 (PMC10936820; doi:10.1371/journal.pone.0298682)
Supplement: S3 Table — (PDF) [file pone.0298682.s003.pdf]

S3 Table. Biogenic amine levels in the brain in workers and gynes (Figure 3)

	Worker (pmol / brain)				
	Dopamine	Serotonin	Tyramine	Octopamine	Protein (mg)
	12.44670697	4.667595177	5.785506975	6.934573529	0.164010133
	11.21789737	3.524038975	3.894572757	7.089595287	0.113994785
	8.7408056	3.576893614	5.677489514	2.125911211	0.103401685
	19.19145954	5.948948986	5.897193394	9.925489209	0.151751469
	18.09489947	5.93859379	12.11508935	11.3760425	0.168423251
	17.32981121	5.84908057	6.918147177	7.441303854	0.133398905
	23.81727647	6.395580092	7.549187976	14.36982359	0.139736346
	15.92849481	4.535384562	4.744361786	6.72069478	0.118189047
	9.646260135	2.897537813	5.688707968	5.610833618	0.10931663
	17.26458132	5.113784314	6.12449694	7.822271531	0.119456535
	15.29591461	3.930725361	6.016148322	7.90832474	0.135088889
	16.49554122	4.139616722	9.604073775	7.619402452	0.191828161
	21.16469911	6.094015311	8.810053767	10.0251042	0.200497143
	17.64324891	4.982941939	6.267645449	9.109260021	0.186809276
	26.9742988	5.669241608	9.800512291	13.97821672	0.221485207
	21.72241238	4.777976404	7.284073983	8.372058915	0.245667106
	12.50662087	1.883247125	2.891160791	7.310837457	0.17950908
	8.590159788	3.845657262	4.047450133	5.584793529	0.201409668
	14.29926265	4.951859735	2.167997288	8.195578237	0.194572597
	11.13815842	4.353261116	5.155327523	6.576104868	0.219628895
	13.40586036	5.171907004	5.133521699	5.146134888	0.218062877
	18.49940097	5.872748947	10.1264861	10.61555561	0.283313654
mean	15.97335323	4.732756201	6.440872953	8.17535958	0.172706879

	Gyne (pmol / brain)				
	Dopamine	Serotonin	Tyramine	Octopamine	Protein (mg)
	25.33828407	9.704875196	12.80378166	15.57935516	0.34177835
	31.51223155	9.83969127	9.930147519	18.44845433	0.325596157
	42.42896416	9.627954341	5.310611451	19.43961846	0.32664017
	23.37911905	6.496219885	11.85631181	9.647587993	0.305237915
	26.07160346	5.489530491	6.552184312	11.57412244	0.136778455
	17.25746792	5.441625453	8.748735008	8.599430462	0.192228576
	21.14798003	7.256007201	8.791464007	10.42822981	0.130965136
	25.40758431	7.087481819	10.48513228	12.82138982	0.194911646
	34.17330367	8.406145066	10.53876626	18.3201818	0.231580275
	23.352695	5.562075254	8.047888154	11.55436574	0.150640986
	18.38515547	6.182937878	11.19847495	9.846345515	0.170764013
	25.26943439	6.590673749	8.722700024	10.66879695	0.151535342
	17.74973629	4.586749815	7.536531226	10.62115041	0.175682976
	17.00218963	6.007272802	8.910975177	10.07245177	0.139461526
	23.20182256	9.048154024	9.947142278	18.43761691	0.210115712
	27.36077737	6.515637297	12.29424745	15.39336111	0.12425746
	18.57732089	1.725652754	6.002159025	8.167780787	0.132306671
	25.300664	3.265559778	7.053997338	9.914084692	0.204302393
	26.94038463	7.946222608	14.66849729	15.75916972	0.155112769
	29.24378174	5.910300184	9.419275736	13.99318162	0.186415257
	33.82145673	4.152288935	8.532649551	11.22381716	0.161820445
	25.72944971	4.671087443	4.629001129	15.03446256	0.157348661
	9.325575824	1.238453024	2.671614866	4.730178927	0.136778455
mean	24.69465141	6.20663462	8.897925587	12.62065801	0.193141711

	Worker (pmol / protein mg)			
	Dopamine	Serotonin	Tyramine	Octopamine
	75.88986592	28.45918789	35.27530212	42.28137263
	98.40711046	30.91403686	34.16448189	62.19227754
	84.53252596	34.59221794	54.90712775	20.55973475
	126.4663841	39.20192029	38.86086533	65.40621508
	107.437063	35.25994029	71.9324039	67.54437053
	129.9096964	43.84654118	51.86059954	55.78234596
	170.444392	45.76890906	54.0245127	102.8352612
	134.7713283	38.37398379	40.14214445	56.86393932
	88.24147045	26.50591978	52.03881599	51.32644176
	144.5260516	42.80874479	51.26966848	65.48215666
	113.2285174	29.09732541	44.53473834	58.5416373
	85.99123905	21.57981762	50.06602649	39.71993697
	105.5611006	30.39452438	43.94104385	50.00123212
	94.44525074	26.67395349	33.55103978	48.76235386
	121.788264	25.59647976	44.24906043	63.11128828
	88.42214481	19.44898725	29.65018033	34.07887635
	69.67124373	10.49109674	16.10593063	40.7268393
	42.65018596	19.09370739	20.09560999	27.72852758
	73.49062956	25.44993391	11.14235674	42.12092743
	50.71353842	19.82098535	23.47290194	29.94189293
	61.47704077	23.71750333	23.54147472	23.5993167
	65.29653877	20.72878899	35.74302181	37.46926938
mean	96.97098099	28.99202298	39.11678669	49.36710062
SE	6.843135626	1.954017818	3.140966671	3.956162532
N	22	22	22	22

Gyne (pmol / protein mg)			
Dopamine	Serotonin	Tyramine	Octopamine
74.13659782	28.395231	37.46223735	45.58321253
96.78318019	30.2205387	30.49835601	56.66054074
129.8951204	29.47572049	16.25829259	59.51386347
76.59310302	21.28248023	38.84285415	31.60678122
190.6119162	40.13446766	47.90362852	84.61948497
89.77576729	28.30809842	45.51214593	44.73544275
161.4779372	55.40411297	67.12827752	79.62599909
130.354367	36.36253629	53.79428306	65.78052186
147.5656927	36.29905472	45.50804798	79.10942251
155.0221868	36.92272216	53.42429304	76.70134194
107.6641098	36.20749923	65.57865874	57.66054175
166.7560452	43.49265095	57.56214948	70.40467783
101.0327622	26.10810638	42.8984721	60.45634402
121.9131194	43.07476757	63.89558077	72.2238748
110.4240247	43.06271979	47.34125872	87.74982481
220.1942427	52.43658837	98.94172487	123.882792
140.4110669	13.04282495	45.36550548	61.73370324
123.8392936	15.98395268	34.52723799	48.52652266
173.6825712	51.22868115	94.5666649	101.5981455
156.8744008	31.70502393	50.52845935	75.06457272
209.0060786	25.65985358	52.7291192	69.35969762
163.5187073	29.68622294	29.41875124	95.54871599
68.18015164	9.054445169	19.53242461	34.58277779
135.4657584	33.19775215	49.53123581	68.81429573
8.761580018	2.514249843	4.149402444	4.521378655
23	23	23	23
