# Supplementary material for: Physiological specialization of the brain in bumble bee castes: Roles of dopamine in mating-related behaviors in female bumble bees
Source: PLoS One. 2024 Mar 13;19(3):e0298682. doi: 10.1371/journal.pone.0298682 (PMC10936820; doi:10.1371/journal.pone.0298682)
Supplement: S4 Table — (PDF) [file pone.0298682.s004.pdf]

S4 Table. Relative expression levels of genes encoding dopamine receptors in the brain in workers and gynes (Figure 4)

Gene	*BigDop1*		*BigDop2*		*BigDop3*		*BigDopEcR*	
Caste	Worker	Gyne	Worker	Gyne	Worker	Gyne	Worker	Gyne
	1.558779	1.425076	1.123117	1.033072	1.04635	0.750111	1.115878	1.184436
	1.636884	1.452424	1.267529	0.912396	1.035079	0.826975	1.319153	1.20182
	1.51535	1.785728	1.210562	1.346595	1.046837	1.119465	1.132997	1.686634
	1.730607	1.16324	1.426432	0.769639	1.22666	0.826181	1.73043	1.083423
	1.498703	1.169623	0.995794	0.965016	0.701146	0.801171	0.9194	0.949739
	1.79098	0.888759	1.536818	0.627775	1.271237	0.54803	1.220836	0.642255
	1.702884	1.63937	1.418228	1.481018	1.276974	1.1805	1.460608	1.264377
	1.092297	1.568363	0.812105	1.160085	0.70445	0.848067	0.81168	1.56196
	1.666785	1.483306	1.246012	0.949686	1.146772	0.863397	1.471844	1.258768
	1.695883	1.178506	1.176125	0.905362	0.810955	0.658937	1.051914	0.875301
mean	1.588915	1.37544	1.221272	1.015064	1.026646	0.842284	1.223474	1.170871
SE	0.062786	0.085523	0.067762	0.080826	0.069448	0.059868	0.087896	0.097748
N	10	10	10	10	10	10	10	10
