# Supplementary material for: Physiological specialization of the brain in bumble bee castes: Roles of dopamine in mating-related behaviors in female bumble bees
Source: PLoS One. 2024 Mar 13;19(3):e0298682. doi: 10.1371/journal.pone.0298682 (PMC10936820; doi:10.1371/journal.pone.0298682)
Supplement: S5 Table — (PDF) [file pone.0298682.s005.pdf]

S5 Table. Data of behavioral activities in workers with dopamine injections (Figure 5)

Locomotor activity

	Not cooled			Cooled		
	Control	10^{-3}M DA	10^{-2}M DA	Control	10^{-3}M DA	10^{-2}M DA
	0	8	50	0	21	0
	0	0	79	0	0	0
	4	0	0	0	0	1
	0	0	60	0	0	0
	0	0	0	0	0	0
	0	23	72	0	0	0
	0	0	0	0	0	0
	50	0	0	0	53	103
	0	0	34	1	0	0
	0	0	0	0	0	35
	16	0		23		126
	0			0		
				0		
mean	5.833333	2.818182	29.5	1.846154	7.4	24.09091
SE	4.231615	2.14399	10.52959	1.764486	5.479659	13.92412
N	12	11	10	13	10	11

Light avoidance

Not cooled			Cooled		
Control	10^{-3}M DA	10^{-2}M DA	Control	10^{-3}M DA	10^{-2}M DA
900	723	683	900	53	900
900	900	139	900	900	900
4	900	900	900	900	900
900	900	802	900	900	900
900	900	900	900	900	900
900	261	193	900	900	900
900	900	900	900	900	900
462	900	900	900	833	778
900	900	553	900	900	900
900	900	900	900	900	44
757	900		872		348
900			900		
			900		
776.9167	825.8182	687	897.8462	808.6	760.9091
79.45434	58.7071	94.42116	2.153846	84.21918	87.36105
12	11	10	13	10	11

Proportion of flying individuals

	Not cooled	Cooled	Total
Control	8.333333	7.692308	8
10^{-3}M DA	9.090909	10	9.52381
10^{-2}M DA	20	36.36364	28.57143
