# Supplementary material for: Physiological specialization of the brain in bumble bee castes: Roles of dopamine in mating-related behaviors in female bumble bees
Source: PLoS One. 2024 Mar 13;19(3):e0298682. doi: 10.1371/journal.pone.0298682 (PMC10936820; doi:10.1371/journal.pone.0298682)
Supplement: S6 Table — (PDF) [file pone.0298682.s006.pdf]

S6 Table. Data of behavioral activities in gynes with flupentixol injections (Figure 6)

Locomotor activity

	Not cooled			Cooled		
	Control	10^{-3}M Flu	10^{-2}M Flu	Control	10^{-3}M Flu	10^{-2}M Flu
	38	0	0	0	0	0
	0	0	0	49	95	0
	111	0	24	38	104	5
	0	0	0	0	90	1
	72	0	0	0	0	0
	49	0	0	0	25	0
	0	0	0	57	0	9
	0	0	0	24	0	16
	0	66	0	0	0	34
	33	0	0	1	34	0
	0	0		0	36	0
	0			13	45	0
	0				0	
mean	23.3076923	6	2.4	15.1666667	33	5.41666667
SE	9.92819387	6	2.4	6.20341222	11.0029134	2.97326046
N	13	11	10	12	13	12

Light avoidance

Not cooled			Cooled		
Control	10^{-3}M Flu	10^{-2}M Flu	Control	10^{-3}M Flu	10^{-2}M Flu
766	900	900	900	900	900
900	900	900	803	505	900
302	900	869	842	649	55
900	900	900	900	693	12
636	900	900	900	900	900
752	900	900	900	146	900
900	900	900	546	900	37
900	900	900	198	900	815
900	541	900	900	900	708
73	900	900	900	802	900
900	900		900	677	900
900			879	285	900
900				900	
748.3846	867.363636	896.9	797.333333	704.384615	660.583333
73.91328	32.6363636	3.1	61.8434403	70.2787069	110.219578
13	11	10	12	13	12

Proportion of flying individuals

	Not cooled	Cooled	Total
Control	30.7692308	33.3333333	32
10^{-3}M Flu	18.1818182	46.1538462	33.3333333
10^{-2}M Flu	0	16.6666667	9.09090909
