# Supplementary material for: Physiological specialization of the brain in bumble bee castes: Roles of dopamine in mating-related behaviors in female bumble bees
Source: PLoS One. 2024 Mar 13;19(3):e0298682. doi: 10.1371/journal.pone.0298682 (PMC10936820; doi:10.1371/journal.pone.0298682)
Supplement: S7 Table — (PDF) [file pone.0298682.s007.pdf]

S7 Table. Number of mated and unmated individuals (Figure 7)

	Number of individuals (%)	
	Control	Fulpentixol
Unmated	4 (36.364)	9 (81.818)
Mated	7 (63.636)	2 (18.182)
N	11	11
