# Supplementary material for: Physiological specialization of the brain in bumble bee castes: Roles of dopamine in mating-related behaviors in female bumble bees
Source: PLoS One. 2024 Mar 13;19(3):e0298682. doi: 10.1371/journal.pone.0298682 (PMC10936820; doi:10.1371/journal.pone.0298682)
Supplement: Attachment — Submitted filename: Response to Reviewers.docx [file pone.0298682.s008.docx]

**Response to reviewers’ comments**

Black: reviewers’ comments

Red: our replies to reviewers’ comments

Line numbers in our replies indicate those in the revised manuscript with track changes.

**Reviewer #1**: This manuscript studies the role of dopaminergic signalling in the regulation of caste specific behaviors in workers and gynes of bumble bees Bombus ignitus. The authors quantified biogenic amine levels (including dopamine) and expression levels of dopamine receptor genes (BigDop1 to 3 and BigDopEcr) in entire bee brains and related differences in these parameters to behavioral differences (flight and motor activity, light avoidance, mating acceptance). The main findings reported indicate that dopamine levels are higher in 8-day old gynes compared to 8-day workers and that this difference correlates with a higher flight and motor activity as well as with decreased light avoidance in gynes compared to workers. In parallel, a decrease in brain expression of BigDop1 was observed in gynes but not in workers. No differences were found for the other genes. Finally, a pharmacological approach was used to enhance dopamine levels in workers and to reduce them in gynes via the use of the dopamine receptor antagonist flupentixol. Enhancing dopamine levels increased motor and flight activity of 7-9 day old workers; reducing them in gynes of the same age reduced flight activity and mating acceptance.

The manuscript reports interesting data but has at the same time some methodological issues, which raise doubts about the soundness of some of its conclusions. These issues are detailed below.

Thank you very much for your comments on our manuscript and providing useful information. As your comments, several descriptions of methodology were not sufficient and luck of explanation in the previous manuscript. Therefore, in the revised manuscript, we have added more explanations with a new figure (please see below).

1) Measurement of biogenic amines

First of all, it is unclear whether the measurements were performed on a single brain basis, and thus the values reported are the mean values of several individual brains, or whether they were obtained in homogenates of multiple (how many?) brains. This is not specified. Second, reporting HPLC values for entire brains may be misleading as an increase in a specific brain region may go hand-by-hand with a decrease in another brain region with a different role in mediating the same behavior. The result, in this case, would be a ‘no-change’ situation as one would compensate the other and thus, the entire dynamic variation would be lost. While important, massive changes in biogenic amine level could be indeed detected using the approach chosen, fine variations on brain region basis, which could be more informative, are definitely overlooked. The authors should be aware of this situation and discuss it properly. For a different, brain-region based approach see, for instance, [1, 2].

In this study, each sample for the measurement of biogenic amines was taken from a single brain. Since the brain volumes differed largely between workers and gynes, the biogenic amine level was then normalized by an amount of protein in the brain. In the revised manuscript, we have added descriptions regarding the sample taken from a single brain in the text (lines 124-125, 126 and 145).

This study determined biogenic amine levels and expression levels of dopamine receptor genes in the whole brain and examined the effects of dopamine-related drug injections. In terms of behaviors, the whole brain acts as a functional unit in insects, integrating multimodal sensory inputs detected at several sense organs and generating motor outputs to drive several effector organs. This study thus adopted a whole-brain approach and unified the analytical levels among experiments. Detailed analyses based on specific brain regions may be more informative than the whole-brain approach; however, degradation of biogenic amines or mRNA and loss of these substances from the cut surface of the brain during dissection could occur in a brain-region-based approach. We therefore considered that a whole-brain approach was a suitable first step, and further studies should be conducted in specific brain regions.

In the revised manuscript, we have added the above discussion with several citations reporting biogenic amine levels and gene expression levels in specific brain regions suggested by the reviewer (lines 407-422).

In Sasaki and Nagao (2001) (J. Insect Physiol. vol. 47, p1205-1216), the dopamine levels at different brain regions have been compared between reproductive and normal workers in the honey bee. Higher dopamine levels were detected in all brain regions in the reproductive workers, suggesting that the released dopamine defused to brain regions with and without dopaminergic neurons. In *B. ignitus*, dopamine may also spread throughout the brain, but the levels in specific brain regions should be determined and compared between castes in future studies.

2) Measurement of gene expression levels
   The same criticism applies to the approach used to quantify gene expression levels. In this case homogenates of two brains were used but no effort was made to section different brain regions and quantify gene expression levels on brain-region basis. This is particularly worrying as gene expression is known to change in different ways in different brain regions, according to their role and implication in several behaviors. It is not surprising, therefore, that no changes were detected for the majority of genes analyzed. The approach chosen may in fact be hiding significant changes in different regions according to behavioral state and caste, which are overlooked by this procedure. This analysis should be reconsidered.
   For a different, brain-region based approach see, for instance, [3-6].

We partly agree with your comments on the fine approach to determine expression of dopamine receptor genes in specific brain regions. However, we selected the whole brain approach for the quantifications of dopamine levels, gene expression levels and bioassays by drug injections, and unified the same analytical levels among experiments. Even though we could determine the higher expression levels of the dopamine receptor gene in the specific brain region, we could not assay the local effects of dopamine by drug injections in a whole body. Furthermore, the target neurons may extend the axon from one compartment to the others. Messenger RNA of the receptor gene may present largely in soma of neurons, whereas the receptor proteins may distribute in membrane of the dendrites distant from the soma. So, the approach of specific brain regions is not perfect, probably both approaches would be needed.

In the revised manuscript, we discussed the reason why we adopted the whole brain approach in this study and necessity of the brain-region based approach in future. Since these discussions contained the measurement of biogenic amine levels, we combined discussions of biogenic amine levels and expression levels of receptor genes in the same sentence in the text (lines 407-422).

3) Pharmacological manipulation of dopamine levels
The authors chose to increase dopamine levels in workers via abdominal injection of dopamine, and to decrease dopamine levels in gynes via abdominal injection of flupentixol, an antagonist of dopamine receptors.
It is difficult to understand why this partial approach was selected instead of having used the double approach (increase and decrease) both in workers and in gynes. The authors should have injected dopamine and flupentixol in individuals of each caste. In this way, testing for necessity and sufficiency of dopaminergic signalling in the behaviors observed would be possible. Currently, the manuscript seems to provide an incomplete and partial analysis in each caste even if some interesting differences were found. It would be desirable to complete the missing approach in each caste (flupentixol in workers and dopamine in gynes) to provide a complete and accurate picture of the role of dopamine in caste-related behavior. One could for instance show that flight, motor activity and mating acceptance is enhanced in a precocious way in younger gynes.

The experiment was designed so that females with lower behavioral activity were injected with dopamine, and females with higher behavioral activity were injected with a dopamine receptor antagonist. Based on comparisons of behavioral activity between castes, workers were injected with dopamine and gynes with a dopamine receptor antagonist. This methodology could have high detectability on the behavioral experiments. If we inject flupentixol in workers with lower locomotor and flying activities, we might not be able to detect decreased activities.

In the revised manuscript, we have added the above reason in Materials and methods (lines 199-203).

Minor remarks
Introduction
The authors discuss the role of biogenic amines, and more specifically of dopamine, in the regulation of bee behavior (lines 30 -40). Yet, in doing this they reduce this discussion to their own work (6 from 9 references), omitting thereby significant findings on the role of dopamine in bees. This needs to be corrected to achieve a fairer introduction. The role of dopaminergic signalling in appetitive motivation [7, 8], as well as in aversive signalling [9, 10], among others, needs to be mentioned.

As reviewer’s comments, we reduced the discussion about our own work, deleted 4 citations of our papers and added one review paper [Sasaki et al., 2021] to be a fairer introduction. We also added other roles of dopamine in sugar responsiveness [Sheiner et al., 2002], memory retrieval in appetitive context [Mercer and Menzel, 1982], appetitive motivation [Huang et al., 2022; Dong et al., 2023] and aversive learning [Vergoz et al., 2007; Mustard et al., 2012] in Introduction (lines 34-41) and Discussion (lines 450-456).

Lines 62-66: the mention to honey bees is not justified as the manuscript does not provide any data on honey bees. Moreover, the discussion of comparable honey bee findings is reduced to two lines in the Discussion (lines 405-407), which does not allow appreciating the level of similarity between the present findings and those obtained in honey bees.

We have deleted the descriptions compared with honey bees and rewritten the sentences in Introduction (lines 67-71), Discussion (lines 445-449) and Conclusion (lines 476-477).

Materials and Methods
Line 71: Referring to a work by Asada and Ono that readers cannot necessarily consult immediately is not helpful to understand the procedures used in this work. The authors should describe their procedures appropriately.

We have deleted the citation of Asada and Ono (2000) and added explanations about the nest box and food for maintain the nest with bees (lines 75-81).

Line 88: same as above. Moreover, in Sasaki et al there is no proper illustration of the experimental setup used, and this is the critical point to understand the behavioral experiments on locomotor activity. I strongly recommend including a scheme of the behavioral ring-shaped chamber to understand what was quantified here.

There was an illustration of the experimental setup as a supplementary figure in Sasaki et al (2020). However, since this supplementary figure may not be easily accessible, we have made an illustration of experimental set up as Figure 1 and deleted the citation of Sasaki et al. (2020) (lines 97-98, 106-112 and 114-115).

Lines 189-192: Repetition of lines 181-183. Please check for coherence.

We have deleted the description and added the following text “using the same procedures as for the behavioral experiments above.” (lines 214-217).

Line 304: should read “gynes” instead of workers; flupentixol was not assayed in workers, as indicated by the methods section and by the caption of Fig. 5.

This was our mistake. “gynes” was correct. Thank you for your pointing out. We have corrected it (line 330).

Line 310: same as above; gynes instead of workers.

This was also a careless mistake. Thank you. We have corrected it (line 335).

Line 313: “to the injected flupentixol”, and not dopamine.

We have corrected as “to the injected flupentixol” (line 338).

Discussion
Expand to include more relevant information on findings related to the role of dopamine in social bees.

We have added more discussion of other dopaminergic roles including sugar responsiveness, memory retrieval in appetitive context, appetitive motivation, and aversive learning in workers in honey bees. Dopamine effects on the defense of territory and colony in ants have also added in Discussion (lines 450-456). Although these roles of dopamine have been investigated in workers, they may potentially contribute to caste-specific behavior.

References
1. Schulz DJ, Pankiw T, Fondrk MK, Robinson GE, Page RE. Comparisons of juvenile hormone hemolymph and octopamine brain Titers in honey bees (Hymenoptera: Apidae) selected for high and low pollen hoarding. Annals of the Entomological Society of America. 2004;97(6):1313-9. doi: Doi 10.1603/0013-8746(2004)097[1313:Cojhha]2.0.Co;2. PubMed PMID: WOS:000225335000022.
2. Nouvian M, Mandal S, Jamme C, Claudianos C, d'Ettorre P, Reinhard J, et al. Cooperative defence operates by social modulation of biogenic amine levels in the honey bee brain. Proc Biol Sci. 2018;285(1871):20172653. Epub 2018/01/26. doi: 10.1098/rspb.2017.2653. PubMed PMID: 29367399; PubMed Central PMCID: PMCPMC5805953.
3. Iino S, Shiota Y, Nishimura M, Asada S, Ono M, Kubo T. Neural activity mapping of bumble bee (Bombus ignitus) brains during foraging flight using immediate early genes. Sci Rep. 2020;10(1):7887. doi: 10.1038/s41598-020-64701-1.
4. Yamane A, Kohno H, Ikeda T, Kaneko K, Ugajin A, Fujita T, et al. Gene expression and immunohistochemical analyses of mKast suggest its late pupal and adult-specific functions in the honeybee brain. PLoS One. 2017;12(5):e0176809. Epub 2017/05/05. doi: 10.1371/journal.pone.0176809. PubMed PMID: 28472083; PubMed Central PMCID: PMCPMC5417555.
5. Kaneko K, Suenami S, Kubo T. Gene expression profiles and neural activities of Kenyon cell subtypes in the honeybee brain: identification of novel 'middle-type' Kenyon cells. Zoological Lett. 2016;2:14. Epub 2016/08/02. doi: 10.1186/s40851-016-0051-6. PubMed PMID: 27478620; PubMed Central PMCID: PMCPMC4967334.
6. Geng H, Lafon G, Avarguès-Weber A, Buatois A, Massou I, Giurfa M. Visual learning in a virtual reality environment upregulates immediate early gene expression in the mushroom bodies of honey bees. Commun Biol. 2022;5(1):130. doi: 10.1038/s42003-022-03075-8.
7. Dong S, Gu G, Lin T, Wang Z, Li J, Tan K, et al. An inhibitory signal associated with danger reduces honeybee dopamine levels. Curr Biol. 2023;33. doi: 10.1016/j.cub.2023.03.072.
8. Huang J, Zhang Z, Feng W, Zhao Y, Aldanondo A, Sanchez MGdB, et al. Food wanting is mediated by transient activation of dopaminergic signaling in the honey bee brain. Science. 2022;376(6592):508-12. doi: doi:10.1126/science.abn9920.
9. Mustard JA, Vergoz V, Mesce KA, Klukas KA, Beggs KT, Geddes LH, et al. Dopamine Signaling in the Bee. In: Galizia CG, Eisenhardt D, Giurfa M, editors. Honeybee Neurobiology and Behavior: A Tribute to Randolf Menzel. Dordrecht: Springer Netherlands; 2012. p. 199-209.
10. Vergoz V, Roussel E, Sandoz JC, Giurfa M. Aversive learning in honeybees revealed by the olfactory conditioning of the sting extension reflex. PLoS One. 2007;2(3):e288. Epub 2007/03/21. doi: 10.1371/journal.pone.0000288. PubMed PMID: 17372627; PubMed Central PMCID: PMC1810431.

**Reviewer #2**: In order to investigate the roles of dopamine in regulating caste-specific behaviors and mating-related behaviors in bumble bees, the authors examined caste differences in behaviors, biogenic amine levels, and analyzed the effects of dopamine-related drugs on bumble bee behavior. The results showed that the locomotor and flight activities were significantly higher in 8-day-old gynes and light avoidance was significantly lower in 4–8-day-old gynes than in same-aged workers. Brain levels of dopamine and octopamine were significantly higher in 8-day-old gynes than in same-aged workers, but tyramine and serotonin levels did not differ between the castes. Dopamine significantly enhanced locomotor and flight activities in 7–9-day-old workers, whereas the dopamine receptor antagonist flupentixol inhibited flight activity and mating acceptance in same-aged gynes. These results suggested that dopamine played important roles in gyne-specific behavior in bumble bees. These results might be interesting for scientists in this field. The manuscript could be improved with minor revision.

Thank you very much for your comments on our manuscript. We have corrected the manuscript followed by your comments.

1.The English of the whole paper should be polished by native English experts carefully.

We have asked the English editing by a native English expert for polishing. We believe that the revised manuscript has been more improved.

2.The references should be corrected according to the requirement of this journal.

Thank you for pointing out. We have corrected the reference style according to the journal requirements.
