# Supplementary material for: Physiological specialization of the brain in bumble bee castes: Roles of dopamine in mating-related behaviors in female bumble bees
Source: PLoS One. 2024 Mar 13;19(3):e0298682. doi: 10.1371/journal.pone.0298682 (PMC10936820; doi:10.1371/journal.pone.0298682)
Supplement: Attachment — Submitted filename: Response to Reviewers.docx [file pone.0298682.s009.docx]

**Response to reviewer’s comments**

Black: comments from reviewer #1

Red: our replies to reviewer’s comments

Line numbers in our replies indicate those in the revised manuscript with track changes.

Reviewer #1: I thank the authors for having addressed all my comments. Although I may not necessarily subscribe some arguments justifying the whole brain approach for molecular analyses related to behavior, I understand the arguments provided by the authors and I consider that they are valid. I will not, therefore, further insist on this point, as the results presented in the manuscript are valuable and worth being published.
I have only some very minor remarks that can be easily corrected by the authors in order to better convey their message and ideas.

Thank you very much for your useful comments and suggestions for revising the text into better English. In the revised manuscript, we have corrected all the points you pointed out.

I am listing these corrections below:

Line 77: “…was dived IN TWO sections…”

We have corrected the sentence as “Each wooden box was divided in two sections” (lines 72-73).

Line 107: “… can walk freely within the ANNULAR circuit (gray area, WHICH WAS COVERED BY A transparent cover sheet. THE SHEET was separated….”
In the description of the experimental setup as well in the new figure itself; the height of the setup containing the bumble bee is missing. This is important to appreciate how were the flight attempts when the red cover was suppressed.

We have corrected the sentence as you suggested (line 103) and added information of the height of the annular circuit in text (lines91-92) and Figure 1B. Since the test chamber was made of stainless steel, we have added this information in the text (line 91 and 100). In figure 1, we have corrected from “Male” to “Female”.

Line 215: “… behavioral experiments DESCRIBED above”.

We have added “described” in the sentence (line 210).

Line 410: “…detected BY several sense organs…”.

We have corrected as “by” in the sentence (line 403).

Line 412: “ … unified analytical levels among experiments”. I don’t understand what is meant here. Please use another wording to make the idea clearer.

We have deleted this phrase from the text (line 405).

Line 416-417: “…a suitable first step THAT SHOULD BE FOLLOWED BY A BRAIN-REGION BASED APPROACH.”

We have corrected this sentence (lines 409-411).

Line 450: “Dopamine PLAYS OTHER ROLES IN THE BEHAVIOR OF SOCIAL INSECTS”. IN HONEY BEES, IT MEDIATES A DECREASE OF SUCROSE RESPONSIVENESS [11; BUT SEE HUANG ET AL, SCIENCE 2022, FIG. 3], AFFECTS MEMORY RETRIEVAL IN AN APPETITIVE CONTEXT [12, 13] , REGULATES APPETITIVE MOTIVATION [13,14] AND MEDIATES AVERSIVE REINFORCEMENT SIGNALING IN AVERSIVE LEARNING [15,16]. IN ANTS, DOPAMINE IS RELEVANT FOR THE DEFENSE OF THE TERRITORY OR COLONY [35].”

We have rewritten these sentences as you suggested (lines 439-445).
